# A new strategy to increase RNA editing at the Q/R site of GluA2 AMPA receptor subunits by targeting alternative splicing patterns of ADAR2

**DOI:** 10.1016/j.jneumeth.2021.109357

**Published:** 2021-12-01

**Authors:** Helena Chaytow, Ilda Sethw Hassan, Sara Akbar, Linda Popplewell, George Dickson, Philip E. Chen

**Affiliations:** Centres of Gene and Cell Therapy and Biomedical Sciences, Department of Biological Sciences, Royal Holloway University of London, Egham, Surrey, UK

**Keywords:** Neurodegeneration, Antisense oligonucleotides, AMPARs, ADAR2, Amyotrophic lateral sclerosis

## Abstract

**Background:**

The GluA2 subunit of AMPA receptors (AMPARs) undergoes RNA editing at a specific base mediated by the enzyme ADAR2, changing the coded amino acid from a glutamine to arginine at the so-called Q/R site, which is critical for regulating calcium permeability. ADAR2 exists as multiple alternatively-spliced variants within mammalian cells with differing editing efficiency.

**New method:**

In this study, phosphorodiamidate morpholino oligomers (PMOs) were used to increase Q/R site editing, by affecting the alternative splicing of *ADAR2*.

**Results:**

PMOs targeting the *ADAR2* pre-mRNA transcript successfully induced alternative splicing around the AluJ cassette leading to expression of a more active isoform with increased editing of the GluA2 subunit compared to control.

**Comparison with existing method(s):**

Previously PMOs have been used to disrupt RNA editing via steric hindrance of the GluA2 RNA duplex. In contrast we report PMOs that can increase the expression of more catalytically active variants of ADAR2, leading to enhanced GluA2 Q/R RNA editing.

**Conclusions:**

Using PMOs to increase Q/R site editing is presented here as a validated method that would allow investigation of downstream cellular processes implicated in altered ADAR2 activity.

## Highlights

• Aberrant RNA editing has been linked to a number of neurodegenerative diseases.
• Phosphorodiamidate morpholino oligomers (PMOs) were targeted to ADAR2 pre-mRNA.
• These PMOs increased expression of ADAR2 isoforms with higher editing efficiency.
• These PMOs significantly increased Q/R editing in HeLa and SH-SY5Y cell lines.

## Graphical Abstract

**Figure:**
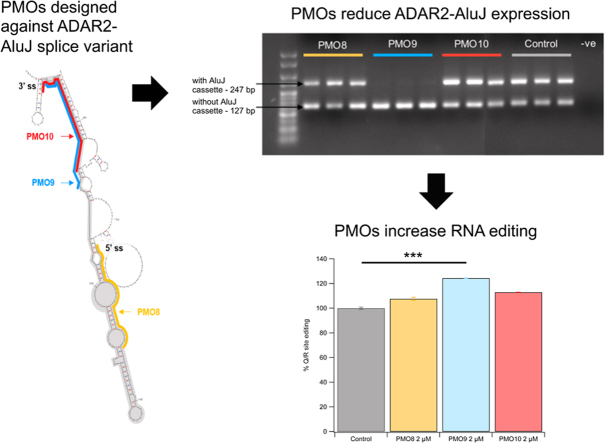

## 1 Introduction

The process of RNA editing describes the alteration of bases in the RNA transcript, and can include insertions, deletions or base changes (Behm et al., 2016). RNA-seq data has revealed millions of editing events in the transcriptome, mostly in non-coding regions and particularly in Alu repeats, with varying levels of editing at each site (Tan et al., 2017). Due to its abundance in Alu elements and neuronal transcripts, RNA editing is considered a key component in the development and diversity of CNS and dysregulation of A-to-I editing is found in a variety of neurological diseases (Hwang et al., 2016, Moore et al., 2019, Tran et al., 2019). The Adenosine Deaminase Acting on RNA (ADAR) family of enzymes catalyse the deamination of adenosine to form the base inosine, otherwise known as A-to-I editing, in double-stranded RNA transcripts (Behm et al., 2016). The edited nucleoside inosine is recognised as guanosine by translational machinery due to the similarity in structure and subsequently changes specific amino acids in coding sequences (for example AMPAR GluA2 subunits and 5-HT_2C_ receptor subunits) (Behm et al., 2016); this base change can also alter the function of non-coding RNAs (Yang et al., 2013). The two main vertebrate ADARs, ADAR1 and ADAR2, edit distinct pools of target transcripts that have some degree of overlap but not complete redundancy (for example the Q/R site in the GluA2 transcript is solely edited by ADAR2). Moreover, ADAR2 exists in multiple isoforms depending on alternative splicing events (Rueter et al., 1999), and inclusion of an Alu sequence (termed the AluJ cassette) after exon 5 in the mRNA can decrease editing efficiency (Gerber et al., 1997).

Phosphorodiamidate morpholino oligomers (PMOs) are neutrally-charged synthetic oligonucleotides with a modified backbone protecting them from degradation by nucleases. PMOs can be designed to target any DNA or RNA sequence, and have been used to manipulate RNA splicing (Havens and Hastings, 2016). It has previously been shown that a PMO directly targeted to the GluA2 RNA transcript disrupts RNA secondary structure and reduces Q/R site editing (Mizrahi et al., 2013, Penn et al., 2013). However, since disrupted editing has been reported in ALS patients, PMOs designed to increase Q/R site editing could be therapeutically relevant. Here we assessed the use of PMOs to manipulate A-to-I editing at the Q/R site of GluA2 subunits by directly targeting the ADAR2 transcript to induce alternative splicing and expression of more catalytically active forms of ADAR2 (AluJ cassette lacking). PMOs were assessed for their AluJ cassette skipping efficiencies, and were tested for their effects on Q/R site editing in a heterologous cell model (HeLa) and in human neuroblastoma cell lines endogenously expressing *GRIA2* RNA for the GluA2 subunit (SH-SY5Y).

## 2 Materials and methods

### 2.1 Design of PMOs

PMOs were designed based on pre-mRNA transcript sequences from ensembl.org (*Gria2*: ENSMUSG00000033981; *ADARB1*: ENSG00000197381). The secondary structure of pre-mRNA transcripts was predicted using MFold (mfold.rna.albany.edu). Intermolecular binding energies of PMOs were calculated using the SOligo function of SFold (sfold.wadsworth.org), and internal binding was calculated using OligoEvaluator (Sigma; www.oligoevaluator.com).

### 2.2 PMO names and sequences

PMOs targeting the *ADAR2 transcript were titled “ADAR2 + a + b” where a and b delineate the sequence targeted in the AluJ cassette. The PMO sequences are as follows:*

PMO8(ALUJ+1+25)-5’CCAGCCTGGGTGTAAGAGCGAGACC3’,

PMO9(ALUJ+93+117)-5’TAGTCCCAGCTCCTTGGAAGGTTGA3’,

PMO10(ALUJ+99+120)-5’CTGTAGTCCCAGCTCCTTGGAAGGT3’.

PMOs were synthesised by GenetoolsLLC and reconstituted at 1 mM in ddH_2_O and stored at +4 °C.

### 2.3 Cell culture

HeLa and SH-SY5Y cells (Sigma) were cultured in Dulbecco’s modified Eagle’s medium (DMEM) (Sigma) supplemented with 10(v/v)% foetal bovine serum (Invitrogen) and penicillin/streptomycin (Invitrogen). Cells were kept at 37 °C and subcultured every 3–4 days or 80% confluent.

### 2.4 Transfection

0.5 µg of plasmid containing a short section of GluA2 intron/exon 18 (B13 GluA2 minigene) (Higuchi et al., 1993) was transfected into HeLa cells using Lipofectamine 2000 (Invitrogen) at a ratio of 2:1 (volume of lipofectamine µl: amount of DNA µg). All transfections were performed following manufacturer’s instructions. 0.03–5 µM of PMOs were transfected into HeLa and SH-SY5Y cells using 6 µM Endoporter (Gene Tools) and were incubated at 37 °C for 24 h before total RNA extraction. Flow cytometry was performed using a BD FACSCantoII.

### 2.5 RNA extraction and RT-PCR

RNA extraction from transfected cells was performed using the ReliaPrep RNA Cell Miniprep system (Promega) according to manufacturer’s instructions. A 0.5 µg sample of total RNA was used for subsequent nested RT-PCR using the GeneScript RT-PCR system (GeneSys Ltd). Primers for both rounds of the nested PCR were targeted to exons 5 and 7, which surround the AluJ cassette (ADAR2 Forward Outer, ATCCATCTTTCAGAAATCAGAGC, ADAR2 Reverse Outer, TTTGGTCCGTAGCTGTCCTC, ADAR2 Forward Inner, AGGCTGAAGGAGAATGTCCA, ADAR2 Reverse Inner, TTGCTTTACGATTTGGGTGTC). RT-PCR was performed using outer forward and outer reverse primers in a MJ Research PTC-200 Thermal Cycler under the following conditions: 45 °C for 30 min (reverse transcription reaction), 92 °C for 2 min (Initial denaturation), 10 cycles of 92 °C (Denaturation) for 30 s, 62 °C (Annealing) for 30 s and 68 °C (Extension) for 45 s, then 25 cycles of 92 °C (Denaturation) for 30 s, 60 °C (Annealing) for 30 s and 68 °C (Extension) for 45 s with an added 5 s per cycle, then a final extension step at 68 °C for 10 min. For second round PCR with inner forward and inner reverse primers the following conditions were used: 95 °C (Initial denaturation) for 3 min, 18 cycles of 94 °C (Denaturation) for 30 s, 60 °C (Annealing) for 30 s and 72 °C (Extension) for 45 s followed by a final extension step of 72 °C for 10 min.

### 2.6 Densitometry

Images of gels were taken under ultraviolet light with 0.16 ms acquisition ensuring that exposure times avoided saturated pixels and using the Ebox VX2 imaging system (PeqLab) and saved as TIFF files. Using the primers indicated, the 247 bp product contains the AluJ cassette, while the 127 bp band lacks the AluJ cassette. The fluorescent intensity of the DNA bands in each lane was quantified using densitometric analysis using ImageJ. The peaks of intensity for each band were identified and the corresponding area measured for quantifying AluJ insertion and AluJ exclusion and “skipping percentages” were determined by dividing the “skipped band” with total fluorescence (total DNA).

### 2.7 Q/R site editing analysis

For HeLa cells, RNA editing was semi-quantified from RT-PCR products using primer sequences for murine premRNA GluA2 (B13 Forward, ATCTGGATGTGCATTGTGTTTGCC, B13 Reverse, ACAAATGGTCGGCAGCTGCTGCTGA). This generated a 314 bp amplicon containing the A-to-I editing site. Digestion with the restriction endonuclease *Bbv*I only digested cDNA copies containing the unedited sequence to produce fragments of 195 bp and 113 bp. The undigested full length band (314 bp) represented edited cDNA. Fluorescence of each band was semi-quantified using densitometric analysis using ImageJ. The fluorescence of the edited fragment was divided by the total fluorescence of all three bands (total DNA) to give a percentage of edited DNA fragments for each sample. The level of editing induced by the different PMOs was expressed relative to editing seen in control cells.

For SH-SY5Y cells a nested PCR was performed to detect endogenous *GluA2* mRNA using the following primers: Human GRIA Forward Outer, CAAAGCCCTTCATGAGCCTC, Human GRIA Reverse Outer, CCATGAATGTCCACTTGAGACC, Human GRIA Forward Inner, GCCTCAGAAGTCCAAACCAG, Human GRIA Reverse Inner, CCATGAATGTCCACTTGAGACC. This generated a full length amplicon of 322 bp (RNA edited) and two *BbvI* digested bands of 238 bp and 85 bp (unedited). In both HeLa and SH-SY5Y experiments the amplicons were generated using the PCR protocol described in Section 2.4.

### 2.8 5HT2C receptor PCR

RNA was isolated as described in 2.4 and RT-PCR was performed using the following primers flanking the edited region: Human HTR2C forward, TGTCCCTAGCCATTGCTGATATGC, Human reverse, GCAATCTTCATGATGGCCTTAGTC, 95 °C for 3 min (Initial denaturation), 35 cycles of 95 °C for 30 s (Denaturation), 55 °C for 30 s (Annealing) and 72 °C for 30 s (Extension) and a final extension step of 72 °C for 2 min.

For the second round PCR, the following inner primers were used, HTR2C forward (nested), CCTGTCTCTCCTGGCAATCC, HTR2C reverse (nested), TCATGATGGCCTTAGTCCGC and produced a 197 bp amplicon, 95 °C for 3 min (Initial denaturation), 35 cycles of 95 °C for 30 s (Denaturation), 55 °C for 30 s (Annealing) and 72 °C for 30 s and a final extension step of 72 °C for 2 min.

### 2.9 Sequencing

GluA2 or HTR2C amplicons were purified using a QIAquick PCR purification kit (Qiagen) and 2 ng/µl was used for sequencing reactions (Eurofins). Quantification of base editing was estimated using EditR (http://baseeditr.com).

### 2.10 Statistical tests and curve fitting

All comparisons were made using one or two-way ANOVAs followed by Bonferroni post-hoc analysis. IC_50_s were calculated by fitting the Hill equation using Igor Pro 6.37 (WaveMetrics, Inc., Lake Oswego, OR, USA).

## 3 Results

### 3.1 Design of PMOs for AluJ exon skipping

**Fig. 1:**
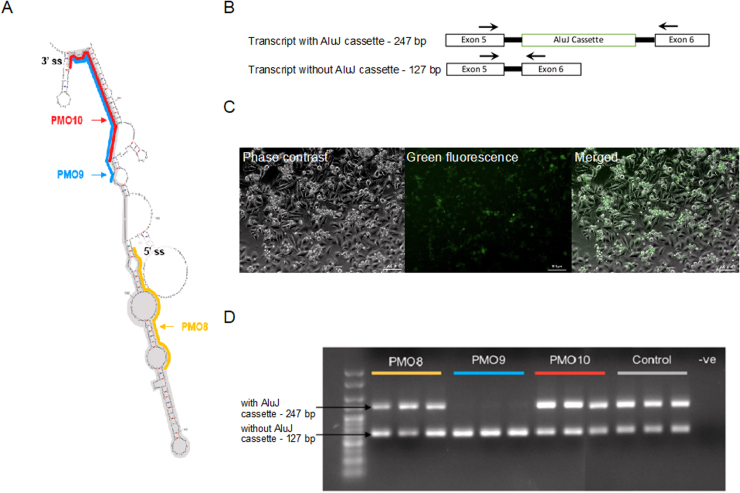
PMOs targeting the AluJ cassette for exon skipping and their effectiveness in excluding the AluJ cassette from *ADAR2* transcripts. A. RNA secondary structure predicted by MFold of the AluJ cassette (shaded in grey) and neighbouring introns (1412 bp) and locations of PMOs. “5’ or 3’ ss” = 5’ and 3’ splice sites flanking the exon. B. Primer design for assessment of exon skipping. Arrows indicate primer placement on transcripts with or without the AluJ cassette, accounting for the difference in product size. C. Phase contrast and fluorescent images displaying transfection of 5 µM of a fluorescently tagged 3’end PMO in HeLa cells. Scale bar 100 µm. D. PCR analysis of cDNA extracts from PMO-treated HeLa cells (transfected with the B13 GluA2 minigene), showing AluJ cassette exclusion following treatment with 2 µM per PMO compared to control. (For interpretation of the references to colour in this figure legend, the reader is referred to the web version of this article.)

Our strategy for influencing RNA editing focused on increasing the catalytic efficiency of the ADAR2 enzyme. Previous work has shown that certain alternatively spliced variants of human *ADAR2* contain a 120 bp sequence (AluJ) that can reduce editing efficiency by 50% (Gerber et al., 1997). Therefore, removal of this AluJ region by promoting alternative splicing could improve editing efficiency. In order to promote exon exclusion, or exon skipping, from an RNA transcript, antisense oligonucleotides are often designed to target exon splice enhancer (ESE) sites or splice junctions (Havens and Hastings, 2016). The human splice finder web tool (www.umd.be/HSF3/) was used to predict where splicing factors would bind in the sequence surrounding the AluJ cassette. These results showed a cluster of ESE binding sites at the 3’ end of the AluJ cassette and clusters of exon splice silencer (ESS) binding sites on either side of the exon (Supplementary Fig. 1). Based on this analysis, the PMOs should be designed to target the 3’ end of the exon. Another consideration in the design of antisense oligonucleotides is the total binding energy between the transcript and the PMO, taking into account any possible self-complementarity of the oligonucleotide (Popplewell et al., 2009). The binding energy calculations were predicted using OligoEvaluator and SFold and the most energetically favourable position for PMO target sequence is at the 3’ splice site of the AluJ exon, with a total binding energy of −6.9 kcal/mol (Supplementary Fig. 2). Targeting the 5’ splice site of the AluJ cassette was calculated to have a positive binding energy of 1.4 kcal/mol, indicating that energy would have to be put into the system for the PMO to bind. The final consideration was the location of the target sequence in the secondary structure of the AluJ sequence. Using MFold to predict the *ADAR2* secondary structure, the 5’ splice site is within a region of double-stranded RNA and is not the ideal target sequence (Fig. 1A). At the 3’ splice site, each end of the 25 base target sequence falls within a double-stranded region. However, if the sequence is shifted 3 bases upstream of the 3’ splice site, the sequence begins in an open region of RNA (Fig. 1A). Three PMOs were therefore synthesised to target the AluJ cassette, targeting either the 5’ end of the AluJ cassette (PMO8(ALUJ + 1 + 25)), the 3’ end of the AluJ cassette (PMO10(ALUJ + 99 + 120)) or 3 bases upstream of PMO10(ALUJ + 99 + 120) (PMO9(ALUJ + 93 + 117)).

### 3.2 Exon skipping of the AluJ cassette and Q/R editing in HeLa cells

**Fig. 2:**
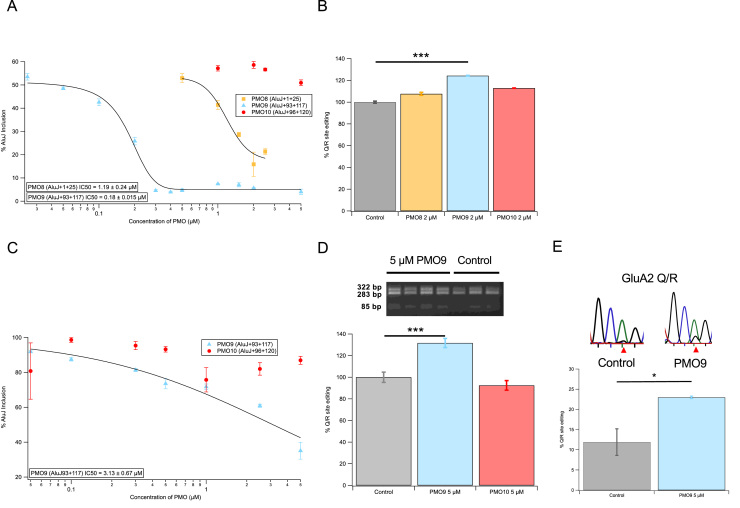
Dose-response analysis of exon skipping efficiencies for targeted PMOs in HeLa and SH-SY5Y cell lines. A. Dose-response curves for PMO8(ALUJ + 99 + 120), 9(ALUJ + 93 + 117) and 10(ALUJ + 99 + 120) and calculated IC_50_s in HeLa cells, n = 6. B) The effects of AluJ cassette skipping induced by 2 µM PMO9(ALUJ + 93 +117) exhibits significant increases in Q/R site editing compared to controls and PMO8(ALUJ + 99 + 120) and 10(ALUJ + 99 + 120) in the HeLa cells. *** p < 0.0005 (ANOVA and Bonferroni post-hoc test), n = 3. C. Dose-response curves for PMO9(ALUJ + 93 + 117) and PMO10(ALUJ + 99 + 120) and calculated IC_50_s in SH-SY5Y cell lines, n = 4. D. Gel images of *Bbv*I digested RT-PCR products from SH-SY5Y cells transfected with and without 5 µM PMO9(ALUJ + 93 + 117). Below, AluJ cassette skipping induced by 5 µM PMO9(ALUJ + 93 + 117) exhibits significant increases in Q/R site editing compared to controls and PMO10(ALUJ + 99 + 120) in SH-SY5Y cell lines. *** p < 0.0005 (ANOVA and Bonferroni post-hoc test), n = 4. E. Example sequencing chromatograms from RT-PCR products from control or 5 µM PMO9 treated SH-SY5Y cells over the GluA2 Q/R edited site (red triangle). * p < 0.05 (ANOVA and Bonferroni post-hoc test), n = 3. (For interpretation of the references to colour in this figure legend, the reader is referred to the web version of this article.)

The PMOs were transfected into HeLa cells and RT-PCR analysis was used to determine the extent of exon skipping (Fig. 1B). Using a fluorescently tagged PMO9(ALUJ + 93 + 117), we visualised PMO transfection using Endoporter in HeLa cells (Fig. 1C). Using flow cytometry we measured a 97.09 ± 0.15% mean frequency of cells positive for fluorescence. Untreated HeLa cells showed inclusion of the AluJ cassette in 62.7 ± 0.97% of total transcripts. 2 µM of PMO8(ALUJ + 1 + 25) targeting the 5’ end of the AluJ cassette showed a moderate but significant effect compared to untreated cells, reducing levels of AluJ cassette inclusion by 15.87 ± 5.21% (p < 0.0001). PMO9(ALUJ + 93 + 117) and PMO10(ALUJ + 99 + 120) target the 3’ end of the AluJ cassette and overlap by 22 of their 25 bases. However, PMO10(ALUJ+99 +120) (targeting the end of the exon) had no significant effect on exon skipping (58.6 ± 1.45%; p = 0.0628) whereas PMO9(ALUJ + 93 + 117), which targets the open structure of the transcript, induced near-complete skipping of the AluJ exon (0.62 ± 0.41% AluJ inclusion; p < 0.0001; Fig. 1D). Dose response curves (0.03–5 µM) for exon exclusion were produced for each PMO, with PMO9(ALUJ + 93 + 117) clearly showing the highest efficiency with a mean IC_50_ of 0.18 ± 0.015 µM. PMO8(ALUJ + 1 + 25) had a calculated mean IC_50_ of 1.19 ± 0.24 µM while PMO10(ALUJ+99 +120) showed little inhibition of exon inclusion (Fig. 2A).

It has previously been reported that ADAR2 isoforms excluding the AluJ cassette are more efficient at A-to-I editing (Gerber et al., 1997) and the PMOs were tested in the B13-HeLa system to assess their effect on Q/R site editing. 2 µM of each PMO was transfected into the B13-HeLa system and Q/R site editing was quantified. Q/R site editing percentages were calculated and normalised to endogenous editing in the B13-HeLa system (Fig. 2B). PMO9(ALUJ + 93 + 117) was the only PMO to have a significant effect on Q/R site editing (p < 0.05), with an increase to 124 ± 1.62% of control. PMO8(ALUJ + 1 + 25) had no significant effect on Q/R site editing (107 ± 1.00% of control) and nor did PMO10(ALUJ + 99 +120) (113 ± 3.93%).

### 3.3 Assessing the effects of PMOs on AluJ cassette exon skipping Q/R site editing in SH-SY5Y cell lines

A number of neuroblastoma-derived cell lines endogenously express Q/R edited GluA2 subunits and we examined the effectiveness of PMO9(ALUJ + 93 + 117) and PMO10(ALUJ + 99 + 120) in SH-SY5Y cell lines. Dose-response curves (0.05–5 µM) for PMO9(ALUJ + 93 + 117) showed a concentration-dependent decrease in AluJ cassette inclusion but the IC_50_ was higher than that seen in HeLa cells (mean IC_50_ = 3.13 ± 0.67 µM) (Fig. 2C). As seen in Fig. 2A, PMO10(ALUJ + 99 + 120) had little effect on AluJ cassette inclusion. Q/R site editing percentages were calculated and normalised to endogenous SH-SY5Y editing and 5 µM PMO9(ALUJ + 93 + 117) displayed significant increases in Q/R editing (131.7 ± 4.2%) compared to PMO10(ALUJ + 99 + 120) or controls (p < 0.0005) (Fig. 2D). Furthermore to confirm editing at the Q/R site, sequencing revealed a rise (23 ± 0.3%) in mean editing following treatment with PMO9(ALUJ + 93 + 117) compared to control cells (11.9 ± 3.3%) (p < 0.05) (Fig. 2E). Therefore PMO9(ALUJ + 93 + 117) can increase RNA editing in both HeLa and SH-SY5Y cell lines compared to PMO10(ALUJ + 99 + 120) or cell-only controls. We also explored whether PMO9(ALUJ + 93 + 117) could also increase RNA editing at other sites and examined editing at the 5HT_C_-receptor transcript (Supplemental Fig. 3). Cell-only controls showed mean editing at site A as 15.25 ± 1.7%, n = 4 whereas this was slightly increased (but insignificant, p = 0.27) in PMO9(ALUJ + 93 + 117) treated cells with a mean editing of 27.3 ± 10.5%, n = 4.

## 4 Discussion

This study successfully used PMOs to target the alternative splicing pattern of the ADAR2 enzyme in both HeLa and SH-SY5Y cell lines and consequently demonstrated an increase in RNA editing at the Q/R site of the GluA2 subunit. PMOs were successfully used to target the AluJ cassette in the *ADAR2* transcript, leading to skipping of the AluJ cassette. The PMOs tested in this study had different effects on exon skipping, with 0.3 µM PMO9(ALUJ + 93 + 117) treatment showing near-complete exclusion of the AluJ cassette in HeLa cells and 5 µM PMO10(ALUJ + 99 + 120) showing no effect. This may be caused by positioning PMO9(ALUJ + 93 + 117) within an open region of RNA which could improve RNA binding compared to PMO10(ALUJ + 99 + 120). This difference in efficacy highlights the importance of target sequence in PMO design and the effect of small changes in PMO sequence on splicing. Removal of the AluJ cassette from the ADAR2 transcript then led to an increase in Q/R site editing following treatment with PMO9(ALUJ + 93 + 117) compared to control; this supports earlier observations that inclusion of the AluJ cassette reduces the catalytic activity of human ADAR2 (Gerber et al., 1997) by potentially impairing ADAR2-RNA substrate binding (Filippini et al., 2018).

### 4.1 Conclusion

Here we demonstrate the use of PMOs at a new target, the ADAR2 RNA transcript, for exon skipping. Both human and rat ADAR2 isoforms containing the nucleotide insertion within exon 5 show high expression within the brain (Gerber et al., 1997, Rueter et al., 1999) but their role in neuronal function and disease remains unclear. This could be investigated through use of specific cell-penetrating peptides coupled to our PMOs (Zou et al., 2013). Aberrant editing has been found in a variety of neurological diseases including amyotrophic lateral sclerosis (ALS) and autism spectrum disorder (Moore et al., 2019, Tran et al., 2019) and investigating the contributions of different ADAR2 RNA isoforms using PMOs to manipulate alternative splicing would allow us to understand the regulatory mechanisms of RNA editing.

## Author contributions

PEC, LP and GD conceived and designed the experiments; HC, ISH and SA performed the experiments; HC, ISH, SA and PEC analysed the data; All authors contributed to the writing and approved the final draft.

## Declarations of interest

None.
