# Supplementary material for: A new strategy to increase RNA editing at the Q/R site of GluA2 AMPA receptor subunits by targeting alternative splicing patterns of ADAR2
Source: J Neurosci Methods. 2021 Dec 1;364:109357. doi: 10.1016/j.jneumeth.2021.109357 (PMC8573265; doi:10.1016/j.jneumeth.2021.109357)
Supplement: Supplementary file 1 — Supplementary material [file mmc1.docx]

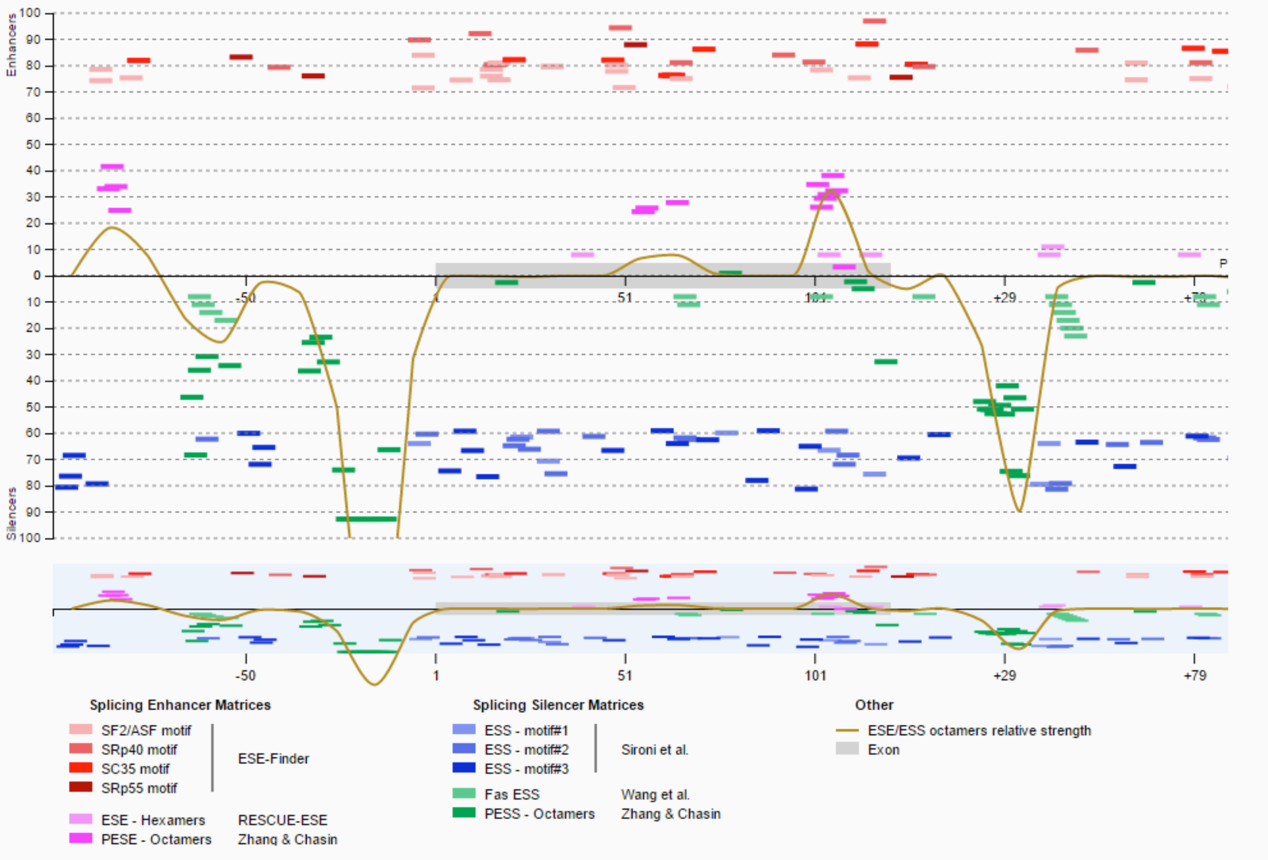


**Supplemental Figure 1** – Output of Human Splice Finder (www.umd.be/HSF3/) showing locations of exon splice enhancer and silencer binding sites surrounding the AluJ cassette of ADARB1. Sequence runs 5’ to 3’ along the x-axis with the grey box indicating the 120 bases of the AluJ cassette. Boxes above the x-axis (in red and pink) indicate sequences associated with splice enhancers while boxes below (in blue and green) represent motifs for exon silencers.


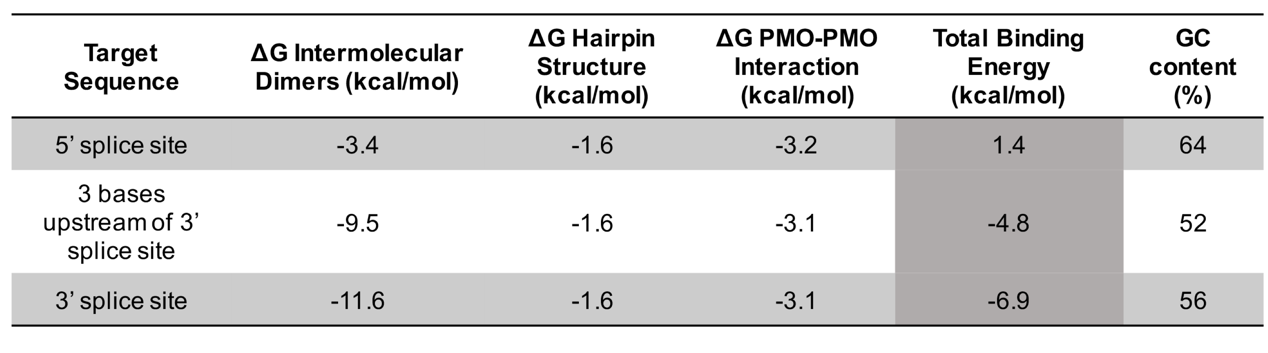


**Supplemental Figure 2**– Binding energies of sequences at each splice site and targeting the open region of RNA structure 3 bases upstream of the 3’ splice site (see Supplemental Figure 1, position 99-120). These values were used to calculate the energy needed for two antisense oligonucleotides to overcome any internal secondary structure. The OligoEvaluator programme provided PMO-PMO interaction and Hairpin structure binding energies for a given sequence. These energies were subtracted from the Intramolecular binding energies obtained from Sfold (sfold.wadsworth.org) to produce the Total binding energy between the antisense oligonucleotide and the RNA transcript.

5-HT_C_ (site A)

Control

PMO9

**Supplemental Figure 3** – RNA editing from example sequencing chromatograms from RT-PCR products from control or 5 µM PMO9(ALUJ+93+117) transfected SH-SY5Y cells at the 5-HTC transcript (Site A, red triangle). p=0.27 (ANOVA and Bonferroni post-hoc test), n=4.
